# Inhibition of HCV by the serpin antithrombin III

**DOI:** 10.1186/1743-422X-9-226

**Published:** 2012-10-02

**Authors:** Mohammed Asmal, Michael Seaman, Wenyu Lin, Raymond T Chung, Norman L Letvin, Ralf Geiben-Lynn

**Affiliations:** 1 Division of Viral Pathogenesis, BIDMC, Boston, MA 02215, USA; 2 Harvard Medical School, Boston, MA 02215, USA; 3 Gastrointestinal Unit, Massachusetts General Hospital, Boston, MA 02114, USA

**Keywords:** Antithrombin III, Hepatitis C virus, OR6 replicon cells, NFκB, P38 MAPK, ERK1/2

## Abstract

**Background:**

Although there have been dramatic strides made recently in the treatment of chronic hepatitis C virus infection, interferon-α based therapy remains challenging for certain populations, including those with unfavorable IL28B genotypes, psychiatric co-morbidity, HIV co-infection, and decompensated liver disease. We have recently shown that ATIII, a serine protease inhibitor (serpin), has broad antiviral properties.

**Results:**

We now show that ATIII is capable of inhibiting HCV in the OR6 replicon model at micromolar concentrations. At a mechanistic level using gene-expression arrays, we found that ATIII treatment down-regulated multiple host cell signal transduction factors involved in the pathogenesis of cirrhosis and hepatocellular carcinoma, including Jun, Myc and BMP2. Using a protein interactive network analysis we found that changes in gene-expression caused by ATIII were dependent on three nodes previously implicated in HCV disease progression or HCV replication: NFκB, P38 MAPK, and ERK1/2.

**Conclusions:**

Our findings suggest that ATIII stimulates a novel innate antiviral host cell defense different from current treatment options.

## Background

Antiviral treatment of hepatitis C virus (HCV) is aimed at persistent eradication of the virus, as measured in sustained virological response (SVR). SVR rates are high with current treatment options, a combination of peg-interferon-apha (IFN-α)–ribavirin and direct-acting antiviral agent (DAA) but HCV patients infected with HIV and/or other co-morbidities might benefit less from the new treatment options [1].

HCV infection is currently one of the most clinically relevant co-morbidities in the HIV population; it affects 15–30% of the 1 million HIV-positive patients in the US [2]. Furthermore, progression to end-stage liver disease occurs six times faster in co-infected patients [3-6], with decompensated cirrhosis being one of the primary causes of hospitalization and death in this population [4,6-8]. Treatment of HCV infection in HIV-positive patients has a lower success rate [9-13] and an increased risk of hepatotoxicity from antiretroviral drugs compared to HIV-negative patients [14-16]. Because of an increased risk of life-threatening complications during peg-IFN-α–ribavirin therapy, patients with hepatic decompensation are not typically candidates for this therapy unless easy access to orthotopic liver transplantation is available [17,18]. Furthermore, since the antiviral activity of IFN-α is mediated, at least in part, through the cytokine network, immunological abnormalities, such as those that often result from HIV infection, reduce IFN-α efficacy. This loss of efficacy has the result of higher treatment failure in HIV/HCV coinfected patients when compared to HCV monoinfected patients [19-22].

The effective treatment of HIV in persons with advanced cirrhosis may be challenging due to cirrhosis-induced alterations in the hepatic metabolism of antiretroviral drugs and the potential for increased risk of drug-induced liver injury. To prevent possible liver toxicity, drug doses may be reduced and certain otherwise preferred drugs may be avoided [23,24]. Reducing antiretroviral doses and hence plasma concentrations, however, may also lower the barrier to the emergence of drug-resistant HIV. Thus, effective therapy to eliminate HCV is necessary to optimize therapy for HIV.

We are investigating here antithrombin III (ATIII), a member of the serine protease inhibitor protein family (serpins), as its anti-inflammatory and anticoagulant activities were found to decrease liver damage [25]. Serpins participate in the early innate immune response to viral infection [26] and they simultaneously possess broad-spectrum anti-viral and anti-inflammatory capabilities. In the case of HIV infection, serpins reportedly interfere with viral replication at both the entry and the reverse transcription stages [27-30]. In particular, the serpins alpha-1 anti-trypsin, the secretory leukocyte protease inhibitor and ATIII have significant *in vitro* ability to inhibit HIV-1, with the latter, ATIII, being the most potent [29-34].

In HCV infections with co-morbidities new drugs with different mechanisms of action other than the DAAs are urgently needed. We hypothesized that the broad immunomodulatory and anti-viral properties of ATIII might extend to other chronic viral infections due to a different mechanism of action, in particular, since a serpin receptor, the LDL receptor-related protein 1 (LRP1), is highly expressed on hepatocytes [34] and was found to block HCV infection [35].

Therefore, we undertook an investigation of whether ATIII has the potential to inhibit HCV replication *in vitro*. We used gene-arrays to probe the molecular mechanisms underlying ATIII’s immunomodulatory and antiviral properties, and uncover the signal transduction pathways that result in inhibition of viral replication.

## Results

### ATIII treatment augments the inhibition of HCV replication by IFN-α

IFN-α is currently part of the standard therapy for chronic HCV infection, in addition to ribavirin and an NS3-4A protease inhibitor. In certain patient subpopulations, this regimen is not always effective or is poorly tolerated. We have previously reported that the serpin ATIII has potent anti-viral activity against HIV [33,34]. We sought to determine whether ATIII might also have activity against HCV since serpin receptors are highly expressed on hepatocytes [36]. We employed the OR6 replicon system [37] expressing full-length genotype 1b virus to assess whether ATIII is capable of inhibiting HCV [38,39]. Although heparin activation augments the anti-HIV activity of ATIII we used unmodified ATIII because heparin activation also increases the off-target effects of ATIII on thrombin. Unmodified ATIII has a demonstrated favorable toxicity profile and has been used in humans for more than 20 years.

**Figure 1:**
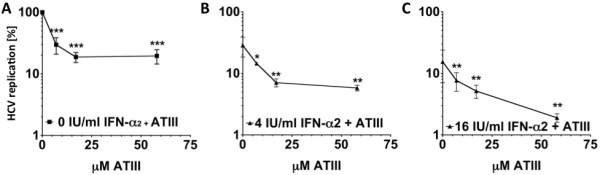
**Additive effect of simultaneous ATIII and IFN-α treatment on HCV replication.** (**A**) Effect of ATIII treatment alone on HCV replication. Significant inhibition is indicated as asteriks in compare to vehicle treated control (***, P<0.001, one-table *T*-test, n=6). (**B**) Additive effect of ATIII on 4 IU/ml IFN-α2 treatment (n=3). (**C**) Additive effect of ATIII on 16 IU/ml IFN-α2 treatment (n=3). Luciferase expression in HCV OR6 replicon cells was used as an indicator of HCV replication after treatment with IFN-α2 and/or ATIII. 5x10^4^ HCV OR6 replicon cells were treated with different concentrations of IFN-α2 and ATIII. Residual luciferase activity as percentage of the vehicle treated control was used to quantify HCV replication. The additive effect of ATIII (7, 17, 58 μM) on different concentrations of IFN-α2 (in *B* and *C*) is shown by significant decrease (*, P<0,05; **, P<0.01; *** P<0.001, unpaired *T*-test) in residual luciferase activity in compare to inhibition of IFN-α2 starting concentration (4, 16 IU/ml without ATIII treatment). Error bars represent standard error of the mean.

We initially explored the effect of ATIII monotherapy on HCV replication. We treated OR6 replicon cells with 7, 17 and 58 μM of ATIII for 48 h. We had previously demonstrated that these concentrations effectively inhibited HIV replication *in vitro*[40]. We quantified viral inhibition as the percentage of residual luciferase activity compared to a vehicle treated control. We observed that ATIII monotherapy inhibited HCV replication in the replicon system in a dose dependent manner, with the lowest dose of 7 μM inhibiting virus 70.2% ±8.8% (p<0.001, n=6) (Figure 1A).

For comparison, we assessed the ability of IFN-α2 monotherapy to inhibit the replicon. We tested doses of 4 and 16 IU IFN-α2, and found 71.4±10.1% and 84.4±8.4% inhibition of HCV, respectively. These results are similar to what has been reported previously [41]. We next sought to determine whether ATIII and IFN-α might have an additive effect on HCV replication. We treated replicon cells with 7, 17 and 58 μM ATIII and with 4 and 16 IU/ml IFN-α2 (Figure 1B/C). We observed an additive effect, as treatment with ATIII significantly decreased HCV replication compared to IFN-α2 monotherapy (P-value of between <0.05 and <0.01). This additive effect was already observed at the lowest dose (7 μM) of ATIII tested (Figure 1). We performed similar experiments using IFN-α5, a different subtype of IFN-α, and confirmed the additive effects of ATIII observed with IFN-α2 (data not shown).

To exclude the possibility that the antiviral effect of ATIII was due to a cytotoxic effect, we assayed for cytotoxicity using Neutral Red and Trypan Blue exclusion staining at the indicated concentrations of drugs. Neither ATIII alone or in combination with IFN-α2 or IFN-α5 showed a cytotoxic effect (data not shown).

### ATIII-induced alterations in gene expression in non-replicon cells

To assess the effect of ATIII treatment on host cell gene expression in the absence of HCV protein expression, we treated Huh7.5. non-replicon cells with the highest concentration of ATIII that would be used in the subsequent gene array experiments: 24 U/ml (58 μM), which is 24-fold the physiologic concentration. We found no significant alterations in expression of genes in the array following ATIII treatment of the non-OR6 replicon cells, demonstrating that, at these concentrations and in the absence of HCV replication, ATIII has no significant effect on expression of our transcriptional pathways of interest. Using Trypan Blue exclusion staining we also found no drug-related cytotoxicity at the concentrations used (data not shown).

### HCV-induced alterations of hepatocyte gene expression

To assess the effect of HCV replication on hepatocyte physiology we compared the transcriptional profile of HCV replicon cells to that of Huh7.5 non-replicon cells using the Transduction Pathfinder RT2 Profiler PCR Assay. Initially, experiments were performed in the absence of exogenous ATIII. We observed substantial differences in the transcription of multiple genes involved in the innate host cell response between cells expressing and not-expressing HCV (Tab. I). Many of these HCV-induced changes have been previously described elsewhere [42-49] confirming the validity of our system. The gene with the greatest increase in expression was Matrix Metallopeptidase 10 (MMP10) (49-fold, P = 0.006), a key mediator in the Jak-Stat pathway and part of the inflammatory response of the host cell against HCV. Additional effects of HCV on host gene expression include activation of p53 and TGF-β pathways: we found a 3-fold (P = 0.03) increase of Cyclin-dependent kinase inhibitor (CDKN) 1A and a 10-fold (P = 0.005) increase of CDKN2B. Other observations from this study that are consistent with previously described associations with HCV include findings of a 9-fold (P = 0.04) increase of Bone morphogenetic protein 4 (BMP4), part of the hedgehog pathway, and a 4-fold (P = 0.03) increase in Heat Shock Protein (HSP) 90AA2, part of the cellular stress response.

### Effect of ATIII on HCV-induced changes in gene expression

We subsequently sought to determine if ATIII might modulate the effects of HCV on host gene expression. We treated replicon cells with 7 μM ATIII, a concentration at which inhibition of HCV replication was observed, and compared gene expression to untreated replicon cells (Table 1). None of the genes affected by HCV expression appeared to be substantially affected by ATIII treatment at this lowest dose.

**Table 1:** Determination of ATIII treatment effect on gene expression in the context of HCV

**Gene**	**Symbol**	**Replicon/Non-replicon Gene expression**		**Replicon/Replicon+7 μM ATIII Gene expression**	
		**Fold change**	**P-value**	**Fold change**	**P-value**
Bone morphogenetic protein 4	BMP4	9.15	0.04	−1.68	0.09
Cyclin-dependent kinase inhibitor 1A	CDKN1A	2.61	0.03	−1.64	0.1
Cyclin-dependent kinase inhibitor 2B	CDKN2B	10.98	0.005	−1.59	0.1
Cytochrome P450, family 19, subfamily A, polypeptide 1	CYP19A1	5.70	0.002	−1.73	0.01
Fatty acid synthase	FASN	4.01	0.01	−2.87	0.9
Heat shock 27kDa protein 1	HSPB1	2.82	0.001	−1.21	0.6
Heat shock protein 90kDa alpha class A member 2	HSP90AA2	4.01	0.03	−1.14	0.6
Interleukin 4 receptor	IL4R	4.76	0.01	−1.79	0.04
Matrix metallopeptidase 10	MMP10	49.29	0.006	−2.19	0.3
Ornithine decarboxylase 1	ODC1	4.49	0.01	1.35	0.4
Retinol binding protein 1	RBP1	2.66	0.045	−1.59	0.2
WNT1 inducible signaling pathway protein 1	WISP1	6.32	0.008	−1.16	0.6

At higher concentrations of ATIII, we found only a modest effect on HCV-induced transcriptional changes (data not shown). There was no ATIII dose-dependent effect on expression of any of the genes in Table I. These results suggest that the mechanism by which ATIII inhibits HCV within 48 h may not involve modulation of the genes influenced by HCV infection.

### ATIII-induced alterations in replicon cell gene expression

HCV infection often leads to chronic hepatitis, cirrhosis, and occasionally to hepatocellular carcinoma [50]. This progression in liver pathology is associated with increased expression in hepatocytes of the transcription factors JUN and MYC, which may play crucial roles in oncogenesis [51,52]. In order to investigate the influence of ATIII on pathways important for HCV disease progression we employed the Transduction Pathfinder RT2 Profiler PCR Assay to quantify the expression of 84 key genes belonging to 18 different regulatory pathways in the presence of different concentrations of ATIII.

To investigate whether the therapeutic use of ATIII might have an influence on gene-expression in OR6 replicon cells, we treated these cells with supra-physiologic concentration of ATIII: 2.4-fold (7 μM), 7-fold (17 μM) and 24-fold (58 μM) blood concentrations. We used supra-physiologic doses of ATIII in part because ATIII is known to accumulate in the liver - a fact which may be of therapeutic advantage.

**Figure 2:**
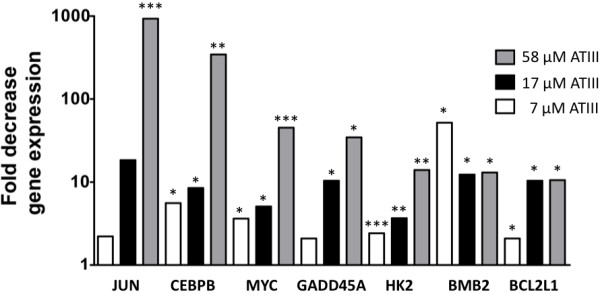
**Key signal transduction pathfinder genes altered after ATIII treatment of HCV OR6 replicon.** Analysis of activated signal transduction pathways in HCV OR6 replicon cells after treatment with ATIII. 5x10^4^ HCV OR6 replicon cells were treated with 7, 17, 58 μM ATIII for 48 h. A real-time PCR expression array was used to analyze the expression of 84 key genes from 18 signal transduction pathways. Asterisks show genes with significant changes (*, P<0,05; **, P<0.01; *** P<0.001) in gene expression compared to vehicle control as calculated by the ΔΔC_t_ method of three independent experiments are shown. All genes are normalized using the GAPDH house-keeping gene. Only genes with more than a 5-fold change in gene-expression are shown.

Treatment of replicon cells with these doses of ATIIII altered expression by more than 5-fold in a group of genes when compared to vehicle treated controls (Figure 2). Interestingly, genes that were most significantly affected were all down-regulated. Among those genes found to be down-regulated following ATIII treatment were JUN and MYC, which are known to be important factors in the pathogenesis of HCV-related hepatocellular carcinoma. We found that these genes were down-regulated in a dose dependent manner, up to 931-fold for JUN (P = 0.0002), and up to 45-fold for MYC (P = 0.0007) at 58 μM.

The next largest decrease in gene expression, up to 346-fold (P = 0.009 at 58 μM), was observed for the transcription factor CAAT/enhancer binding protein (CEBPB), a protein regulated by insulin. Another gene downstream of insulin Hexokinase 2 (HK2), was down-regulated up to 14-fold (P = 0.0039). Growth arrest and DNA-damage-inducible protein (GADD45A), a gene in the p53 pathway, was down-regulated 35-fold at 58 μM (P = 0.0023). Bone morphogenetic protein 2 (BMP2), a gene of the Hedgehog pathway, was down-regulated 13-fold (P = 0.03) at 58 μM. B-cell CLL/lymphoma2-like 1 (BCL2L), a transcript belonging to the Jak/Src pathway, exhibited an approximately 10-fold decrease in expression (Figure 2). Down-regulation of these genes was specific to ATIII treated OR6 cells with ongoing HCV replication, and was not observed in the untreated OR6 replicon, nor in the ATIII-treated Huh7.5 controls suggesting that ATIII induces a specific anti-viral gene program.

### Changes in gene expression when ATIII is used in combination with IFN-α

**Figure 3:**
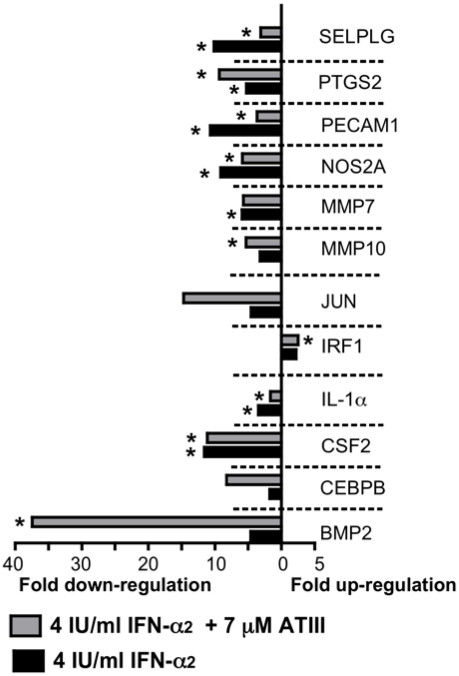
**Down-regulation of key signal transduction pathway genes in HCV OR6 replicon after treatment with IFN-α and ATIII.** 5x10^4^ HCV OR6 replicon cells were treated with 7 μM ATIII/ml and 4 IU/ml IFN-α for 48 h, and RNA was purified. A real-time RT-PCR expression array was used to analyze the expression of 84 key genes from 18 signal transduction pathways. Genes with significant changes (*, P<0.05), in gene expression compared to vehicle control as calculated by the ΔΔC_t_ method of three independent experiments are shown. All genes are normalized using the GAPDH house-keeping gene. Only genes with more than a 2-fold change in gene-expression are shown.

Our data indicate that both IFN-α and ATIII stimulate the host innate antiviral response. We sought to determine whether these proteins engaged redundant cellular pathways, or had non-overlapping mechanisms of action. We applied IFN-α2 in isolation, to measure the gene expression alterations attributable to this drug. The gene expression profile induced by IFN-α monotherapy that we observed was consistent with previous reports [53,54]. At 4 IU/ml, we found Interferon regulatory factor 1 (IRF-1) up-regulated 2-fold. We also identified a set of inflammatory genes that were down-regulated: Colony stimulating factor 2 (CSF2) (inhibited 11.5-fold, P=0.01), IL-1α (inhibited 4-fold, P=0.02), MMP7 (inhibited 6-fold, P=0.04), MMP10 (inhibited 3-fold), Nitric oxide synthase 2A (NOS2A) (inhibited 9-fold, P=0.009), and Prostaglandin-endoperoxide synthase (PTGS2) (5-fold, P=0.01) (Figure 3).

When IFN-α was administered in combination with ATIII, additional genes were significantly altered, potentially explaining the additive antiviral effect of ATIII when added to IFN-α treatment. The most substantially down-regulated gene was BMP2, belonging to the Hedgehog pathway, which was decreased by 37-fold (P=0.04). JUN and PTGS2 both belonging to the Phospholipase C pathway were 14-fold (P=0.08) and 9-fold (P=0.02) down-regulated. CEBPB of the insulin pathway was 8-fold down-regulated (Figure 3).

We repeated these experiments using IFN-α5, to exclude the possibility that our results may have been idiosyncratic to IFN-α2. We observed the same gene expression pattern with IFN-α5 treatment, with or without ATIII treatment (data not shown).

### Network analysis of ATIII-induced interactomes in OR6 replicon cells

To gain further insight into the mechanism of action of ATIII in reducing HCV replication, we performed a biologic network analysis of ATIII treated OR6 replicons. This analysis method complements data generated from our gene arrays by facilitating the recognition of hierarchical gene clusters that might intersect with HCV replication. This software-supported interactome analysis is based on a vast library of gene interactions known to regulate certain pathways and allows to identify pathways effected by a drug.

**Figure 4:**
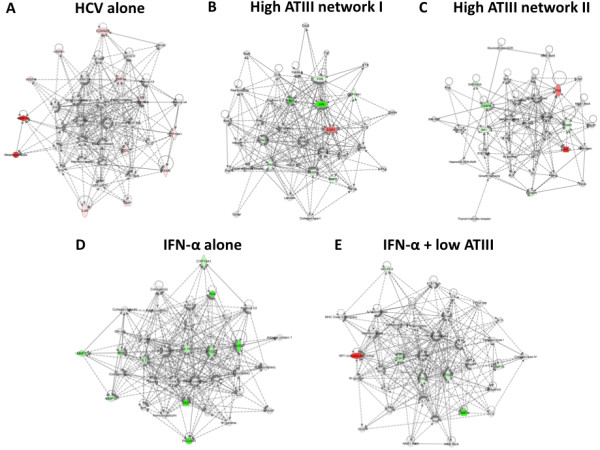
**Interactive network analysis of HCV, ATIII and IFN-α altered hepatocyte genes.** (**A**) Network with nodules and significant genes altered by HCV. (**B and C**) Two highest scoring network (out of three) with nodules affecting genes activated by high dose (58 μM) ATIII treatment of the HCV replicon. (**D**) Network with nodules and significant altered genes after IFN-α treatment of the HCV replicon. (**E**) Network with nodules and significant altered genes after IFN-α and low dose (7 μM) ATIII treatment of the HCV replicon. Pathway analysis was done using Ingenuity Pathways Analysis Software with significant (P<0.05) up-regulated (red) and down-regulated (green) genes indicated. Nodules (double circles) are genes with a reported influence on genes altered by HCV, ATIII or IFN-α. Significance was calculated using the ΔΔC_t_ method for three independent experiments.

We used genes which we had observed to be significantly up-regulated by HCV replication (Table 1) to generate interactomes describing host cell transduction pathways activated by HCV. We identified nodules regulated by ERKs, AKT, PI3K, RAS, NFκB, P38, P38 MAPK and MAPK as all being activated by HCV infection (Figure 4A). We then assessed the influence of treatment with the high dose (58 μM) of ATIII on gene expression to determine which of these HCV nodules were affected by gene expression changes downstream of ATIII. We found that ATIII interacted with two independent networks that were also modulated by HCV. The highest scoring network was mostly dependent on the ERKs (Figure 4B), and the second highest scoring network interfered with NFκB and P38 MAPK (Figure 4C). These results suggested that despite our observation that ATIII and HCV alter the expression of different sets of individual genes, transcriptional programs activated by ATIII may interfere with 3 out of the 6 nodules activated by HCV. We hypothesize that this might be significant enough to counteract some of the pathologic effects of HCV.

We have demonstrated additive activity of IFN-α and ATIII in inhibiting HCV. We thus next sought to determine whether they might exhibit overlapping effects on the HCV interactome. We compared the effect of IFN-α/low ATIII dose therapy to that of IFN-α alone on HCV induced nodules. Treatment with 4 IU/ml IFN-α alone altered three HCV induced nodules: P38 MAPK, MAPK and NFκB (Figure 4D). The addition of low dose ATIII did not alter the number of nodules affected, but did increase the IRF1 response (Figure 4E) which is known to be responsible for some of the HCV inhibition and might be the reason for the additive effect on IFN-α treatment.

## Discussion

The cornerstone of effective therapy for chronic hepatitis C infection has been IFN-α, a critical mediator in the innate immune response to viral infection. Even with the advent of small molecule direct inhibitors of viral enzymatic activity, IFN-α remains important for attaining sustained virologic response, perhaps because of the need to engage host antiviral programs to completely eradicate viral reservoirs. However, interferon-based therapy is not without its shortcomings, including poor tolerability or poor efficacy in certain patient populations.

We now demonstrate that a novel activator of host innate antiviral responses, ATIII, may give insight into adjunctive therapies for HCV that might augment or even replace IFN-α in cases where there are co-morbidities or genetic factors that preclude the use of IFN-α. There is circumstantial evidence that ATIII may play a role in the pathogenesis of HCV infection: low plasma concentrations of ATIII have previously been correlated with progression to chronic hepatitis and cirrhosis [55]. Furthermore, there is a high density of serpin receptors on hepatocytes, suggesting that serpins may have localized effects on hepatic innate immunity [56].

We used the OR6 replicon to probe how ATIII might influence HCV pathogenesis. We demonstrated that ATIII inhibited HCV replication at micromolar concentrations. While this inhibition was not as potent as that of either IFN-α or fluvastatin [41] it was many-fold greater than that of ribavirin [57].

We next investigated the mechanism of ATIII’s anti-HCV activity. After more than 20 years of research, the mechanism of action of IFN-α in inhibiting HCV has only recently been determined. HCV cell-based expression models, as the one used in this study, were used to demonstrate that IFN-α-induced signal transduction through the Jak/STAT pathway was necessary for HCV inhibition [45]. To elucidate the mechanism(s) through which serpins activate the host defense system, we employed the OR6 replicon system, and analyzed changes in gene-expression patterns of 84 key genes representative of 18 different signal transduction pathways. We found that ATIII treatment down-regulated JUN expression. It has previously been shown that the JNK inhibitor, SP600125, interferes with the oncogenic potential of HCV non-structural protein 3 [58]. In addition, we found that ATIII treatment reduced induction of the transcription factor MYC, expression of which has been associated with progression of liver disease to chronic hepatitis, cirrhosis, and hepatocellular carcinoma [52,59]. ATIII treatment also reduced CEBPB, a transcription factor of the CCAAT/enhancer-binding protein class. These transcription factor proteins have been shown to be important for HCV inhibition: the CCAAT/enhancer-binding protein-homologous protein (CHOP) is activated by HCV envelope protein and is associated with disease progression [60]. The mechanism of action of this protein class is not fully understood, but CHOP can sensitize cells to apoptosis by down-regulation of BCL-2 expression, depletion of cellular glutathione, and exaggerated production of reactive oxygen species [61].

Our data also identified a substantial down-regulation of BMP2, a protein that regulates hepcidin [62]. Hepcidin is critical for iron homeostasis and is also a critical host cofactor involved in promoting HCV replication [63]. Chronic HCV infection has been found to be associated with increased serum iron and transferrin saturation as well as hepatic iron accumulation; moreover, hepatic hepcidin mRNA expression is increased in patients with chronic HCV infection [64]. Hepcidin transcription is stimulated by iron overload as well as by inflammation through IL-6 [65], which is elevated in patients with chronic HCV. The identification of hepcidin as a HCV replication cofactor suggests a molecular basis for the well-known clinical association between chronic HCV infection and dysregulation of iron homeostasis. Moreover, it is possible that the up-regulation of hepcidin transcription by IL-6 potentially creates a positive feedback loop between chronic inflammation and HCV replication. Together, these findings suggest that ATIII therapy may reduce the pathogenic impact of HCV infection.

Thus, our data indicate that ATIII targets several genes that are known to promote both liver disease and HCV replication. ATIII treatment may therefore alter the expression of these genes and act to simultaneously slow both HCV replication and ultimately liver degeneration. ATIII’s effect on gene-expression was also observed when replicon cells were co-treated with low concentrations of IFN-α. It was during this dual drug treatment that gene expression of BMP2, CEBPB, and JUN were most dramatically down-regulated.

Protein interactive network analysis demonstrated that the genes that were altered by ATIII treatment were dependent on three nodes: NFκB, P38 MAPK and the ERKs. All of these nodes have been described previously as having a role in HCV replication and HCV-related liver disease [47,66-69] confirming ATIII’s potential to limit HCV’s destruction of the liver. These nodules are also affected in ATIII-mediated inhibition of HIV [40].

Although our replicon model can facilitate identification of substances that affect either viral genomic replication or host cell factors involved in viral genomic replication, it cannot be used to identify substances that alter other stages of the viral life cycle. Therefore, future studies using fully infectious, cell-culture-adapted HCV strains will be required to study other aspects of the HCV life cycle, such as viral entry, uncoating, viron assembly and secretion. Our data identified several genes altered by ATIII that were previously shown to be correlated with HCV disease outcome. This might explain the additive therapeutic effect when ATIII was used in combination with IFN-α. We further found that ATIII’s mechanism of action is most likely multi-faceted, warranting further research into each distinct signaling pathway.

## Material and methods

### Cell culture

OR6 replicon cells were a gift from Dr. Nobuyuki Kato (Okayama University, Japan) and were propagated in Dulbecco’s Modified Eagle’s medium (DMEM) containing 10% fetal bovine serum (FBS) supplemented with 1% penicillin-streptomycin, and 500 μg/ml Geneticin (Invitrogen Corp., Carlsbad, CA). Cells were cultured in a 37°C, 5% CO2-humidified incubator for all experiments. To decrease day-to-day variability in the assay, a large homogenous population of subconfluent cells was passaged so that a similar lot of cells could be used throughout the assay.

### Protein reagents

Clinical grade human ATIII (Talecris, Durham, NC) had a concentration of 6 U/mg and a purity >98%. For ATIII drug combination experiments, recombinant human IFN-α2 and IFN-α5 (PBL Interferon Source, Piscataway, NJ) was used, which had a concentration of 2.38 x 10^8^ and 2.33 x 10^8^ units/mg, respectively, and a purity of > 98%.

### Determination of inhibitory potency

HCV replication inhibition was determined as the percentage of luciferase activity retained by the OR6 replicon after ATIII treatment, compared to a vehicle-treated control [37]. Luciferase activity was measured using the Renilla Luciferase Assay System (Promega, Madison, WI) at 1 sec. intervals using the 1420 Multilabel Counter Victor 3 (PerkinElmer, Waltham, MA). Cell viability was assessed using Neutral Red staining and Tryphan Blue exclusion staining.

### Signal transduction pathway profiling

OR6 replicon cells were harvested after 48 h of ATIII and/or IFN-α treatment and total RNA was recovered using the RNeasy Kit (Qiagen) with an on-column DNAse digest (Qiagen) according to the manufacturer’s protocol. Approximately 100 ng RNA was used for cDNA synthesis using the SuperArray RT^2^ First Strand Kit (SABiosciences, Frederick, MD, cat. no. C-03). cDNA was used for the RT^2^ Profiler PCR Array Human Signal Transduction PathwayFinder (SABiosciences, cat. no. PAHS-014A). The genes that were investigated can be found at http://www.sabiosciences.com/rt_pcr_product/HTML/PAHS-014A.html. Three arrays of three independent experiments were performed for each treatment condition. Relative levels of transcription were determined by using the ΔC_t_ values for each gene obtained by subtracting the mean threshold cycle (C_t_) of the GAPDH housekeeping gene from the C_t_ value of the gene of interest. The average ΔC_t_ value for 3 experiments was calculated, for each gene of interest, and the average normalized transcription was calculated as follows: 2 (−averageΔCt)^-1^. Fold increases of gene transcription, before and after treatment was calculated by dividing the average normalized transcription of each gene in the test sample by the corresponding control. Statistical significance in up- or down-regulation of transcription was determined by Student *T*-test (2-sample, equal variance, 2-tailed distribution), comparing the ΔΔC_t_ (ΔΔC_t_ = ΔC_t_ treated - ΔΔC_t_ control). Significant differences were identified when P was less than 0.05.

### Analysis of protein interactive networks and statistical analysis

Functional analysis of interacting proteins was determined using a commercial System Biology package, Ingenuity Pathways Analysis (IPA 8.0) ( http://www.ingenuity.com) following the application protocols.

### Statistical analysis

The statistical significance of differences between groups was determined using the program GraphPad Prism. A P value of <0.05 was considered statistically significant. Statistical analysis was performed using one table *T*-test or the unpaired *T*-test. Error bars represent standard error of the mean (S. E.).

## Competing interests

The authors declare that they have no competing interests.

## Authors’ contributions

MA analyzed data and wrote the manuscript. MS and WL did experiments. RTC and NLL analyzed data. RGL did experiments, analyzed data and wrote the manuscript. All authors read and approved the final manuscript.

## References

[B1] Naggie S Sulkowski MS Management of patients coinfected with HCV and HIV: a close look at the role for direct-acting antivirals Gastroenterology 2012 142 1324 1334 e1323 10.1053/j.gastro.2012.02.012 22537439 PMC3637982

[B2] Rockstroh JK Influence of viral hepatitis on HIV infection J Hepatol 2006 44 S25 S27 16338020 10.1016/j.jhep.2005.11.007

[B3] Benhamou Y Bochet M Di Martino V Charlotte F Azria F Coutellier A Vidaud M Bricaire F Opolon P Katlama C Poynard T Liver fibrosis progression in human immunodeficiency virus and hepatitis C virus coinfected patients The Multivirc Group. Hepatology 1999 30 1054 1058 10.1002/hep.510300409 10498659

[B4] Martin-Carbonero L Benhamou Y Puoti M Berenguer J Mallolas J Quereda C Arizcorreta A Gonzalez A Rockstroh J Asensi V Incidence and predictors of severe liver fibrosis in human immunodeficiency virus-infected patients with chronic hepatitis C: a European collaborative study Clin Infect Dis 2004 38 128 133 10.1086/380130 14679458

[B5] Martinez-Sierra C Arizcorreta A Diaz F Roldan R Martin-Herrera L Perez-Guzman E Giron-Gonzalez JA Progression of chronic hepatitis C to liver fibrosis and cirrhosis in patients coinfected with hepatitis C virus and human immunodeficiency virus Clin Infect Dis 2003 36 491 498 10.1086/367643 12567308

[B6] Bica I McGovern B Dhar R Stone D McGowan K Scheib R Snydman DR Increasing mortality due to end-stage liver disease in patients with human immunodeficiency virus infection Clin Infect Dis 2001 32 492 497 10.1086/318501 11170959

[B7] Rosenthal E Poiree M Pradier C Perronne C Salmon-Ceron D Geffray L Myers RP Morlat P Pialoux G Pol S Cacoub P Mortality due to hepatitis C-related liver disease in HIV-infected patients in France (Mortavic 2001 study) AIDS 2003 17 1803 1809 10.1097/00002030-200308150-00009 12891066

[B8] Gebo KA Diener-West M Moore RD Hospitalization rates differ by hepatitis C satus in an urban HIV cohort J Acquir Immune Defic Syndr 2003 34 165 173 10.1097/00126334-200310010-00006 14526205

[B9] Gilleece YC Browne RE Asboe D Atkins M Mandalia S Bower M Gazzard BG Nelson MR Transmission of hepatitis C virus among HIV-positive homosexual men and response to a 24-week course of pegylated interferon and ribavirin J Acquir Immune Defic Syndr 2005 40 41 46 10.1097/01.qai.0000174930.64145.a9 16123680

[B10] Micallef JM Kaldor JM Dore GJ Spontaneous viral clearance following acute hepatitis C infection: a systematic review of longitudinal studies J Viral Hepat 2006 13 34 41 10.1111/j.1365-2893.2005.00651.x 16364080

[B11] Dominguez S Ghosn J Valantin MA Schruniger A Simon A Bonnard P Caumes E Pialoux G Benhamou Y Thibault V Katlama C Efficacy of early treatment of acute hepatitis C infection with pegylated interferon and ribavirin in HIV-infected patients AIDS 2006 20 1157 1161 10.1097/01.aids.0000226956.02719.fd 16691067

[B12] Kamal SM Fouly AE Kamel RR Hockenjos B Al Tawil A He Q Khalifa KE Koziel MJ El Naggar KM Rasenack J Afdhal NH Peginterferon alfa-2b therapy in acute hepatitis C: impact of onset of therapy on sustained virologic response Gastroenterology 2006 130 632 638 10.1053/j.gastro.2006.01.034 16530503

[B13] Jaeckel E Cornberg M Wedemeyer H Santantonio T Mayer J Zankel M Pastore G Dietrich M Trautwein C Manns MP Treatment of acute hepatitis C with interferon alfa-2b N Engl J Med 2001 345 1452 1457 10.1056/NEJMoa011232 11794193

[B14] Nunez M Lana R Mendoza JL Martin-Carbonero L Soriano V Risk factors for severe hepatic injury after introduction of highly active antiretroviral therapy J Acquir Immune Defic Syndr 2001 27 426 431 11511818 10.1097/00126334-200108150-00002

[B15] Lana R Nunez M Mendoza JL Soriano V Rate and risk factors of liver toxicity in patients receiving antiretroviral therapy Med Clin (Barc) 2001 117 607 610 11714465 10.1016/s0025-7753(01)72194-x

[B16] Sulkowski MS Thomas DL Chaisson RE Moore RD Hepatotoxicity associated with antiretroviral therapy in adults infected with human immunodeficiency virus and the role of hepatitis C or B virus infection JAMA 2000 283 74 80 10.1001/jama.283.1.74 10632283

[B17] Mauss S Valenti W DePamphilis J Duff F Cupelli L Passe S Solsky J Torriani FJ Dieterich D Larrey D Risk factors for hepatic decompensation in patients with HIV/HCV coinfection and liver cirrhosis during interferon-based therapy AIDS 2004 18 F21 F25 15316334 10.1097/00002030-200409030-00002

[B18] Bani-Sadr F Carrat F Pol S Hor R Rosenthal E Goujard C Morand P Lunel-Fabiani F Salmon-Ceron D Piroth L Risk factors for symptomatic mitochondrial toxicity in HIV/hepatitis C virus-coinfected patients during interferon plus ribavirin-based therapy J Acquir Immune Defic Syndr 2005 40 47 52 10.1097/01.qai.0000174649.51084.46 16123681

[B19] Chung RT Andersen J Volberding P Robbins GK Liu T Sherman KE Peters MG Koziel MJ Bhan AK Alston B Peginterferon Alfa-2a plus ribavirin versus interferon alfa-2a plus ribavirin for chronic hepatitis C in HIV-coinfected persons N Engl J Med 2004 351 451 459 10.1056/NEJMoa032653 15282352 PMC4113392

[B20] Torriani FJ Ribeiro RM Gilbert TL Schrenk UM Clauson M Pacheco DM Perelson AS Hepatitis C virus (HCV) and human immunodeficiency virus (HIV) dynamics during HCV treatment in HCV/HIV coinfection J Infect Dis 2003 188 1498 1507 10.1086/379255 14624375

[B21] Manns MP McHutchison JG Gordon SC Rustgi VK Shiffman M Reindollar R Goodman ZD Koury K Ling M Albrecht JK Peginterferon alfa-2b plus ribavirin compared with interferon alfa-2b plus ribavirin for initial treatment of chronic hepatitis C: a randomised trial Lancet 2001 358 958 965 10.1016/S0140-6736(01)06102-5 11583749

[B22] Fried MW Shiffman ML Reddy KR Smith C Marinos G Goncales FL Jr Haussinger D Diago M Carosi G Dhumeaux D Peginterferon alfa-2a plus ribavirin for chronic hepatitis C virus infection N Engl J Med 2002 347 975 982 10.1056/NEJMoa020047 12324553

[B23] Sulkowski MS Mehta SH Chaisson RE Thomas DL Moore RD Hepatotoxicity associated with protease inhibitor-based antiretroviral regimens with or without concurrent ritonavir AIDS 2004 18 2277 2284 10.1097/00002030-200411190-00008 15577540

[B24] Eron J Jr Yeni P Gathe J Jr Estrada V DeJesus E Staszewski S Lackey P Katlama C Young B Yau L The KLEAN study of fosamprenavir-ritonavir versus lopinavir-ritonavir, each in combination with abacavir-lamivudine, for initial treatment of HIV infection over 48 weeks: a randomised non-inferiority trial Lancet 2006 368 476 482 10.1016/S0140-6736(06)69155-1 16890834

[B25] Miyazaki M Kato M Tanaka M Tanaka K Takao S Kohjima M Ito T Enjoji M Nakamuta M Kotoh K Takayanagi R Antithrombin III injection via the portal vein suppresses liver damage World J Gastroenterol 18 1884 1891 22563168 10.3748/wjg.v18.i16.1884 PMC3337563

[B26] Opal SM Esmon CT Bench-to-bedside review: functional relationships between coagulation and the innate immune response and their respective roles in the pathogenesis of sepsis Crit Care 2003 7 23 38 12617738 10.1186/cc1854 PMC154114

[B27] Congote LF The C-terminal 26-residue peptide of serpin A1 is an inhibitor of HIV-1 Biochem Biophys Res Commun 2006 343 617 622 10.1016/j.bbrc.2006.02.190 16554023

[B28] Congote LF Serpin A1 and CD91 as host instruments against HIV-1 infection: are extracellular antiviral peptides acting as intracellular messengers? Virus Res 2007 125 119 134 10.1016/j.virusres.2006.12.018 17258834

[B29] Shugars DC Sauls DL Weinberg JB Secretory leukocyte protease inhibitor blocks infectivity of primary monocytes and mononuclear cells with both monocytotropic and lymphocytotropic strains of human immunodeficiency virus type I Oral Dis 1997 3 Suppl 1 S70 S72 9456661 10.1111/j.1601-0825.1997.tb00379.x

[B30] McNeely TB Shugars DC Rosendahl M Tucker C Eisenberg SP Wahl SM Inhibition of human immunodeficiency virus type 1 infectivity by secretory leukocyte protease inhibitor occurs prior to viral reverse transcription Blood 1997 90 1141 1149 9242546

[B31] McNeely TB Dealy M Dripps DJ Orenstein JM Eisenberg SP Wahl SM Secretory leukocyte protease inhibitor: a human saliva protein exhibiting anti-human immunodeficiency virus 1 activity in vitro J Clin Invest 1995 96 456 464 10.1172/JCI118056 7615818 PMC185219

[B32] Shapiro L Pott GB Ralston AH Alpha-1-antitrypsin inhibits human immunodeficiency virus type 1 FASEB J 2001 15 115 122 10.1096/fj.00-0311com 11149899

[B33] Elmaleh DR Brown NV Geiben-Lynn R Anti-viral activity of human antithrombin III Int J Mol Med 2005 16 191 200 16012749

[B34] Geiben-Lynn R Brown N Walker BD Luster AD Purification of a modified form of bovine antithrombin III as an HIV-1 CD8+ T-cell antiviral factor J Biol Chem 2002 277 42352 42357 10.1074/jbc.M207079200 12192009

[B35] Agnello V Abel G Elfahal M Knight GB Zhang QX Hepatitis C virus and other flaviviridae viruses enter cells via low density lipoprotein receptor Proc Natl Acad Sci USA 1999 96 12766 12771 10.1073/pnas.96.22.12766 10535997 PMC23090

[B36] Lillis AP Van Duyn LB Murphy-Ullrich JE Strickland DK LDL receptor-related protein 1: unique tissue-specific functions revealed by selective gene knockout studies Physiol Rev 2008 88 887 918 10.1152/physrev.00033.2007 18626063 PMC2744109

[B37] Ikeda M Abe K Dansako H Nakamura T Naka K Kato N Efficient replication of a full-length hepatitis C virus genome, strain O, in cell culture, and development of a luciferase reporter system Biochem Biophys Res Commun 2005 329 1350 1359 10.1016/j.bbrc.2005.02.138 15766575

[B38] Alter MJ Kruszon-Moran D Nainan OV McQuillan GM Gao F Moyer LA Kaslow RA Margolis HS The prevalence of hepatitis C virus infection in the United States, 1988 through 1994 N Engl J Med 1999 341 556 562 10.1056/NEJM199908193410802 10451460

[B39] Blatt LM Mutchnick MG Tong MJ Klion FM Lebovics E Freilich B Bach N Smith C Herrera J Tobias H Assessment of hepatitis C virus RNA and genotype from 6807 patients with chronic hepatitis C in the United States J Viral Hepat 2000 7 196 202 10.1046/j.1365-2893.2000.00221.x 10849261

[B40] Whitney JB Asmal M Geiben-Lynn R Serpin Induced Antiviral Activity of Prostaglandin Synthetase-2 against HIV-1 Replication PLoS One 2011 6 e18589 10.1371/journal.pone.0018589 21533265 PMC3075258

[B41] Ikeda M Abe K Yamada M Dansako H Naka K Kato N Different anti-HCV profiles of statins and their potential for combination therapy with interferon Hepatology 2006 44 117 125 10.1002/hep.21232 16799963

[B42] Hassan M Selimovic D Ghozlan H Abdel-kader O Hepatitis C virus core protein triggers hepatic angiogenesis by a mechanism including multiple pathways Hepatology 2009 49 1469 1482 10.1002/hep.22849 19235829

[B43] Miller K McArdle S Gale MJ Jr Geller DA Tenoeve B Hiscott J Gretch DR Polyak SJ Effects of the hepatitis C virus core protein on innate cellular defense pathways J Interferon Cytokine Res 2004 24 391 402 10.1089/1079990041535647 15296650

[B44] Matsuzaki K Murata M Yoshida K Sekimoto G Uemura Y Sakaida N Kaibori M Kamiyama Y Nishizawa M Fujisawa J Chronic inflammation associated with hepatitis C virus infection perturbs hepatic transforming growth factor beta signaling, promoting cirrhosis and hepatocellular carcinoma Hepatology 2007 46 48 57 10.1002/hep.21672 17596875

[B45] Wohnsland A Hofmann WP Sarrazin C Viral determinants of resistance to treatment in patients with hepatitis C Clin Microbiol Rev 2007 20 23 38 10.1128/CMR.00010-06 17223621 PMC1797633

[B46] Meurs EF Breiman A The interferon inducing pathways and the hepatitis C virus World J Gastroenterol 2007 13 2446 2454 17552028 10.3748/wjg.v13.i17.2446 PMC4146763

[B47] Girard S Vossman E Misek DE Podevin P Hanash S Brechot C Beretta L Hepatitis C virus NS5A-regulated gene expression and signaling revealed via microarray and comparative promoter analyses Hepatology 2004 40 708 718 10.1002/hep.20371 15349911

[B48] Dou J Liu P Wang J Zhang X Effect of hepatitis C virus core shadow protein expressed in human hepatoma cell line on human gene expression profiles J Gastroenterol Hepatol 2006 21 1794 1800 10.1111/j.1440-1746.2006.04380.x 17074016

[B49] Park CY Jun HJ Wakita T Cheong JH Hwang SB Hepatitis C virus nonstructural 4B protein modulates sterol regulatory element-binding protein signaling via the AKT pathway J Biol Chem 2009 284 9237 9246 10.1074/jbc.M808773200 19204002 PMC2666576

[B50] Saito I Miyamura T Ohbayashi A Harada H Katayama T Kikuchi S Watanabe Y Koi S Onji M Ohta Y Hepatitis C virus infection is associated with the development of hepatocellular carcinoma Proc Natl Acad Sci USA 1990 87 6547 6549 10.1073/pnas.87.17.6547 2168552 PMC54573

[B51] Machida K Tsukamoto H Liu JC Han YP Govindarajan S Lai MM Akira S Ou JH c-Jun mediates hepatitis C virus hepatocarcinogenesis through signal transducer and activator of transcription 3 and nitric oxide-dependent impairment of oxidative DNA repair Hepatology 52 480 492 20683948 10.1002/hep.23697 PMC3107125

[B52] Farinati F Cardin R Bortolami M Guido M Rugge M Oxidative damage, pro-inflammatory cytokines, TGF-alpha and c-myc in chronic HCV-related hepatitis and cirrhosis World J Gastroenterol 2006 12 2065 2069 16610058 10.3748/wjg.v12.i13.2065 PMC4087686

[B53] Chung RT Gale M Jr Polyak SJ Lemon SM Liang TJ Hoofnagle JH Mechanisms of action of interferon and ribavirin in chronic hepatitis C: Summary of a workshop Hepatology 2008 47 306 320 18161743 10.1002/hep.22070 PMC2799164

[B54] Castet V Fournier C Soulier A Brillet R Coste J Larrey D Dhumeaux D Maurel P Pawlotsky JM Alpha interferon inhibits hepatitis C virus replication in primary human hepatocytes infected in vitro J Virol 2002 76 8189 8199 10.1128/JVI.76.16.8189-8199.2002 12134024 PMC155162

[B55] Sheikh SMR Viunytska LV Anithrombin III as a criteria marker in chronic liver disease The Internet Journal of Laboratory Medicine 2009 3

[B56] Pizzo SV Serpin receptor 1: a hepatic receptor that mediates the clearance of antithrombin III-proteinase complexes Am J Med 1989 87 10S 14S 2552799 10.1016/0002-9343(89)80524-8

[B57] Tanabe Y Sakamoto N Enomoto N Kurosaki M Ueda E Maekawa S Yamashiro T Nakagawa M Chen CH Kanazawa N Synergistic inhibition of intracellular hepatitis C virus replication by combination of ribavirin and interferon- alpha J Infect Dis 2004 189 1129 1139 10.1086/382595 15031779

[B58] Hassan M Ghozlan H Abdel-Kader O Activation of c-Jun NH2-terminal kinase (JNK) signaling pathway is essential for the stimulation of hepatitis C virus (HCV) non-structural protein 3 (NS3)-mediated cell growth Virology 2005 333 324 336 10.1016/j.virol.2005.01.008 15721365

[B59] Cui J Dong BW Liang P Yu XL Yu DJ Construction and clinical significance of a predictive system for prognosis of hepatocellular carcinoma World J Gastroenterol 2005 11 3027 3033 15918184 10.3748/wjg.v11.i20.3027 PMC4305834

[B60] Chan SW Egan PA Hepatitis C virus envelope proteins regulate CHOP via induction of the unfolded protein response FASEB J 2005 19 1510 1512 16006626 10.1096/fj.04-3455fje

[B61] Nishitoh H Matsuzawa A Tobiume K Saegusa K Takeda K Inoue K Hori S Kakizuka A Ichijo H ASK1 is essential for endoplasmic reticulum stress-induced neuronal cell death triggered by expanded polyglutamine repeats Genes Dev 2002 16 1345 1355 10.1101/gad.992302 12050113 PMC186318

[B62] Babitt JL Huang FW Wrighting DM Xia Y Sidis Y Samad TA Campagna JA Chung RT Schneyer AL Woolf CJ Bone morphogenetic protein signaling by hemojuvelin regulates hepcidin expression Nat Genet 2006 38 531 539 10.1038/ng1777 16604073

[B63] Tai AW Benita Y Peng LF Kim SS Sakamoto N Xavier RJ Chung RT A functional genomic screen identifies cellular cofactors of hepatitis C virus replication Cell Host Microbe 2009 5 298 307 10.1016/j.chom.2009.02.001 19286138 PMC2756022

[B64] Aoki CA Rossaro L Ramsamooj R Brandhagen D Burritt MF Bowlus CL Liver hepcidin mRNA correlates with iron stores, but not inflammation, in patients with chronic hepatitis C J Clin Gastroenterol 2005 39 71 74 15599216

[B65] Nemeth E Rivera S Gabayan V Keller C Taudorf S Pedersen BK Ganz T IL-6 mediates hypoferremia of inflammation by inducing the synthesis of the iron regulatory hormone hepcidin J Clin Invest 2004 113 1271 1276 15124018 10.1172/JCI20945 PMC398432

[B66] Schmitz KJ Wohlschlaeger J Lang H Sotiropoulos GC Malago M Steveling K Reis H Cicinnati VR Schmid KW Baba HA Activation of the ERK and AKT signalling pathway predicts poor prognosis in hepatocellular carcinoma and ERK activation in cancer tissue is associated with hepatitis C virus infection J Hepatol 2008 48 83 90 10.1016/j.jhep.2007.08.018 17998146

[B67] Shrivastava A Manna SK Ray R Aggarwal BB Ectopic expression of hepatitis C virus core protein differentially regulates nuclear transcription factors J Virol 1998 72 9722 9728 9811706 10.1128/jvi.72.12.9722-9728.1998 PMC110482

[B68] Tan SL Nakao H He Y Vijaysri S Neddermann P Jacobs BL Mayer BJ Katze MG NS5A, a nonstructural protein of hepatitis C virus, binds growth factor receptor-bound protein 2 adaptor protein in a Src homology 3 domain/ligand-dependent manner and perturbs mitogenic signaling Proc Natl Acad Sci USA 1999 96 5533 5538 10.1073/pnas.96.10.5533 10318918 PMC21894

[B69] Georgopoulou U Caravokiri K Mavromara P Suppression of the ERK1/2 signaling pathway from HCV NS5A protein expressed by herpes simplex recombinant viruses Arch Virol 2003 148 237 251 10.1007/s00705-002-0925-0 12556990

